# Supplementary material for: How does discharge against medical advice affect risk of mortality and unplanned readmission? A retrospective cohort study set in a large UK medical admissions unit
Source: BMJ Open. 2023 Mar 27;13(3):e068801. doi: 10.1136/bmjopen-2022-068801 (PMC10069606; doi:10.1136/bmjopen-2022-068801)
Supplement: Author's manuscript [file bmjopen-2022-068801.draft_revisions.pdf]

BMJ Open is committed to open peer review. As part of this commitment we make the peer review history of every article we publish publicly available.

When an article is published we post the peer reviewers' comments and the authors' responses online. We also post the versions of the paper that were used during peer review. These are the versions that the peer review comments apply to.

The versions of the paper that follow are the versions that were submitted during the peer review process. They are not the versions of record or the final published versions. They should not be cited or distributed as the published version of this manuscript.

BMJ Open is an open access journal and the full, final, typeset and author-corrected version of record of the manuscript is available on our site with no access controls, subscription charges or pay-per-view fees (http://bmjopen.bmj.com).

If you have any questions on BMJ Open's open peer review process please email info.bmjopen@bmj.com

BMJ Open

Discharge Against Medical Advice is associated with increased mortality in younger patients: a retrospective cohort study.

Journal:	*BMJ Open*
Manuscript ID	bmjopen-2022-068801
Article Type:	Original research
Date Submitted by the Author:	04-Oct-2022
Complete List of Authors:	Alagappan, Anand; Royal Infirmary of Edinburgh, Acute & General Medicine<br>Chambers, Tom; The University of Edinburgh Centre for Discovery Brain Sciences; Western General Hospital, Metabolic Unit<br>Brown, Erik; NHS Lothian, Lothian Analytical Services, Waverley Gate<br>Grecian, Sheila; Edinburgh Royal Infirmary, Diabetes and Endocrinology<br>Lockman, Khalida Ann; The University of Edinburgh, Department of Acute Medicine
Keywords:	INTERNAL MEDICINE, PUBLIC HEALTH, Health economics < HEALTH SERVICES ADMINISTRATION & MANAGEMENT

SCHOLARONE™
Manuscripts

I, the Submitting Author has the right to grant and does grant on behalf of all authors of the Work (as defined in the below author licence), an exclusive licence and/or a non-exclusive licence for contributions from authors who are: i) UK Crown employees; ii) where BMJ has agreed a CC-BY licence shall apply, and/or iii) in accordance with the terms applicable for US Federal Government officers or employees acting as part of their official duties; on a worldwide, perpetual, irrevocable, royalty-free basis to BMJ Publishing Group Ltd ("BMJ") its licensees and where the relevant Journal is co-owned by BMJ to the co-owners of the Journal, to publish the Work in this journal and any other BMJ products and to exploit all rights, as set out in our licence.

The Submitting Author accepts and understands that any supply made under these terms is made by BMJ to the Submitting Author unless you are acting as an employee on behalf of your employer or a postgraduate student of an affiliated institution which is paying any applicable article publishing charge ("APC") for Open Access articles. Where the Submitting Author wishes to make the Work available on an Open Access basis (and intends to pay the relevant APC), the terms of reuse of such Open Access shall be governed by a Creative Commons licence – details of these licences and which Creative Commons licence will apply to this Work are set out in our licence referred to above.

Other than as permitted in any relevant BMJ Author's Self Archiving Policies, I confirm this Work has not been accepted for publication elsewhere, is not being considered for publication elsewhere and does not duplicate material already published. I confirm all authors consent to publication of this Work and authorise the granting of this licence.

For peer review only

Discharge Against Medical Advice is associated with increased mortality in younger patients: a retrospective cohort study.

A Alagappan[1*], TJG Chambers[2,3*], E Brown[4], SM Grecian[3], KA Lockman[1]

[1]Department of Acute Medicine

The Royal Infirmary of Edinburgh and The University of Edinburgh

51 Little France Crescent, Edinburgh EH16 4SA, UK

[2]Centre for Discovery Brain Science, University of Edinburgh, Hugh Robson Building, George Square, Edinburgh, EH8 9XD

[3]Metabolic Unit, Western General Hospital, Crewe Road South, Edinburgh, EH4 2XU

[4]Lothian Analytical Services, NHS Lothian. Waverley Gate, 2-4 Waterloo Place, Edinburgh, EH1 3EG

*These authors contributed equally to this publication

Corresponding author:

Dr. Thomas Chambers- Email: tom.chambers@ed.ac.uk

Keywords: discharge against medical advice

For peer review only

ABSTRACT

Objectives: To assess the frequency of DAMA in a large UK teaching hospital, explore factors which increase the risk of DAMA and identify how DAMA impacts patient risk of mortality and re-admission.

Design: Retrospective cohort study.

Setting: Large acute teaching hospital in the United Kingdom.

Patients: 36,683 patients discharged from the acute medical unit of a large UK teaching hospital between January 1, 2012 and December 31, 2016.

Measurements: Patients were censored on January 1 2021. Mortality and 30-day readmission rates were assessed. Deprivation, age and sex were taken as covariates.

Results: 3% of patients discharged against medical advice. These patients were younger (median age (years) (IQR)): Planned Discharge (PD) 59 (41-77); DAMA 39 (28-51), predominantly of male sex (PD 48%; DAMA 66%), and were of greater social deprivation (in three most deprived quintiles PD 69%; DAMA 84%). DAMA was associated with increased risk of death in patients under the age of 33.3 years (adjusted HR 2.6 (1.2-5.8)) and increased incidence of 30-day readmission (standardized incidence ratio 1.9 (1.5-2.2)).

Limitations: Re-admission to acute hospitals outside of the local health board may have been missed. We were unable to include information regarding co-morbidity or severity of presentation.

Conclusions: These data highlight the vulnerability of younger patients who DAMA, even in a free-at-the-point-of-delivery healthcare setting.

INTRODUCTION

For peer review only - http://bmjopen.bmj.com/site/about/guidelines.xhtml

Accounting for an estimated 1-2% of hospital discharges in the United States (1–4), discharge against medical advice (DAMA) occurs when a patient chooses to leave hospital prior to recommendation from their medical team. Of concern, the Nationwide Inpatient Sample in the US highlighted that the annual prevalence of DAMA has increased by 1.9%/annum between 2002 and 2011 (2). These figures are not unique to the United States; studies in other parts of the world show comparable figures although we could not identify examples in the literature from UK based cohorts (5–9).

Several factors associate with risk of DAMA; with a difference in perceived recovery (compared to the physician) and financial constraints thought to be most likely (2). DAMA is prevalent among vulnerable individuals, particularly those with a pre-existing diagnosis of mental health disorders and substance misuse (10,11).

Patients choosing to leave the hospital against the advice of senior clinicians are at increased risk of significant adverse outcomes. For example in previous studies in the USA and Canada, patients who DAMA are up to 13.4% more likely to be re-admitted within 30 days and up to 0.6% more likely to do die within 30 days (10,11). This is thought to be related to a combination of them missing important diagnostic tests and treatment but may also reflect that underlying sociological, economic and psychological vulnerability contribute to decision making around discharge.

AIMS

We aimed to establish the contemporary incidence of DAMA in a large acute UK teaching hospital in a free-at-the-point-of-delivery health care system, identify demographic factors associated with DAMA, and to explore if DAMA was associated with risk of readmission and

For peer review only - http://bmjopen.bmj.com/site/about/guidelines.xhtml

mortality. We considered that such data would be essential to frame revision of policies regarding the optimal management of patients choosing to discharge against medical advice.

For peer review only

For peer review only - http://bmjopen.bmj.com/site/about/guidelines.xhtml

METHODS

The study was approved by the local Caldicott guardian.

Study population

We conducted a retrospective analysis of all patients who were discharged from the Acute Medical Unit (AMU) at the Royal Infirmary of Edinburgh (RIE) between January 1, 2012 and December 31, 2016. The Royal Infirmary of Edinburgh serves as the largest local hospital for the region of Lothian, UK which has a population of 900,000. The RIE has a mean annual admission rate of 60,000/year and covers a geographical area of 700 square miles. The AMU is the admissions unit through which all adult medical admissions are handled. Patients are either treated and discharged directly or admitted downstream to their appropriate specialty if longer courses of treatment are required.

Data were compiled from electronic patient records; at each discharge, nursing staff document if the discharge was planned, against medical advice or was a mortality.

Outcomes and follow-up

The outcome measures were readmission (within 30 days) and mortality up until the point of censor on the 1st of January 2021.

Data extraction

Data was collected using TRAK (Oracle) information gathering software. The Information Services Division (ISD) of NHS Lothian provided the data linkage between the demographics and morbidity and mortality. Demographic variables included were sex, age, and Scottish Index of Multiple Deprivation (SIMD). Readmission dates were obtained from the electronic health record and mortality data obtained from linkage with National Records of Scotland (12).

For peer review only - http://bmjopen.bmj.com/site/about/guidelines.xhtml

3.8% of patients did not have an SIMD as no fixed address of residence was associated with the admission. These reflect patients with no fixed abode and for simplicity we included them among patients in the most deprived quintile. Patients admitted from the accident and emergency department following accidental or intentional overdose are cohorted into a toxicology bay in the acute medical unit and are given a specific toxicology code. We thus coded patients as either 'general medicine' or 'toxicology'.

Readmissions have been identified as any patient who has an unplanned inpatient stay anywhere in NHS Lothian, in the 30 days following discharge from AMU. Inpatient stays are only counted against the most recent previous stay, so as not to count consecutive stays. Inpatient stays that may have occurred outside of NHS Lothian have not been captured.

Statistical analysis was conducted using *R* version 4.0.0. Raw data are presented with comparisons between DAMA and Planned discharge groups assessed using Pearson's Chi-square test for discrete variables and Mann Whitney U-test for continuous variables. Multiple variable logistic regression was used to assess risk factors for DAMA. Cox proportional hazards modelling for mortality was completed using the *survival* package.(13) For each covariate, we tested proportionality of hazards using Pearson product-moment correlations of Schoenfeld residuals with time: we stratified age, SIMD and length of stay to minimise loss of proportionality. We chose to stratify SIMD class as <4 and ≥4 as the Lothian population is skewed to less deprived quintiles. Length of stay was stratified as <2 days or ≥ 2 days given the left skew of the data (predominant short admissions). We included stratification of the Cox models by year of discharge given the trend for reducing numbers of discharges over the study period. Multiple variable logistic regression was used to assess odds of discharge against medical advice and cause of

For peer review only

For peer review only - http://bmjopen.bmj.com/site/about/guidelines.xhtml

death. Indirect standardised incidence rates were calculated using *the sir* function of the *popEpi* package.(14) Significance was taken as p<0.05. Data are presented as medians with interquartile range.

Patient and Public Involvement

Patients or the public were not involved in the design, conduct or reporting or dissemination plans or our research

RESULTS

There were 37,409 discharges from the acute medical unit between 1st January 2012 and 31st December 2016 inclusive. We excluded 13 patients for whom we had no date of birth. 10,918 discharges were excluded as they were not the first discharge for each patient within the study period; 300 were excluded as the patient had died during the admission. 26,187 first patient discharges were assessed. Of these, there were 25,430 planned discharges, and 757 DAMA (3%).

Patients who DAMA were more likely to be male (48.1% of PD population vs. 65.8% of the DAMA population (X^2 = 92.4, p<0.0001); younger (median age 60 years (PD) vs. 39 years (DAMA); p<0.0001(Mann-Whitney U test); of more deprived SIMD (X^2 = 167.5, p <0.0001); were more likely to have an inpatient stay of under 2 days (96.3% (PD) vs 99.3% (DAMA); X^2 = 19.4, p<0.0001) and were more likely to be discharged from the toxicology unit (18.7% (PD) vs. 37.5% (DAMA); X^2 = 168.1, p <0.0001) (Table 1). The number of discharges from AMU reduced for each study year but the proportion of DAMA did not vary significantly ranging between 2.4% and 3.1%.

	PD (N=25430)	DAMA (N=757)	Total (N=26187)	p value

For peer review only - http://bmjopen.bmj.com/site/about/guidelines.xhtml

SEX				< 0.001
- Male (%)	12228 (48.1%)	498 (65.8%)	12726 (48.6%)	
Age at admission (years)				< 0.001
- Median (IQR)	59 (40-77)	39 (28-51)	59 (40-76)	
SIMD,				< 0.001
- 1 most deprived	6817 (27.2%)	310 (43.1%)	7127 (27.7%)	
- 2	5746 (23.0%)	183 (25.4%)	5929 (23.0%)	
- 3	4765 (19.0%)	112 (15.6%)	4877 (18.9%)	
- 4	4001 (16.0%)	70 (9.7%)	4071 (15.8%)	
- 5 least deprived	3697 (14.8%)	45 (6.2%)	3742 (14.5%)	
Length of stay <2 days				< 0.001
- <2 days	22058 (86.7%)	721 (95.2%)	22779 (87.0%)	
- >2 days	3372 (13.3%)	36 (4.8%)	3408 (13.0%)	
Specialty				< 0.001
- General Medicine	20626 (81.1%)	473 (62.5%)	21099 (80.6%)	
- Toxicology	4804 (18.9%)	284 (37.5%)	5088 (19.4%)	
Age of death, years				< 0.001
- n	5734	68	5802	
- Median (IQR)	84 (75-90)	59 (49-70)	84 (75-90)	

Table 1 Summary of differences in patients with planned discharge (PD) compared to those who discharge against medical advice (DAMA). Difference assessed by Chi² for categorical values, or Mann Whitney U test for continuous variables

Age under 33.3 years, male sex, a more deprived SIMD, discharge from a toxicology admission and, length of stay <2 days were all significantly associated with increased odds of DAMA as assessed by multiple variable logistic regression (Table 2).

Characteristic	OR	95% CI	p-value
SEX			
Female	—	—	
Male	1.90	1.63, 2.23	<0.001
SIMD 2 level			
<4 most deprived	—	—	
≥4 least deprived	0.56	0.45, 0.68	<0.001
Stratified age (years)			
13-33.3	—	—	
33.3-66.6	0.67	0.56, 0.79	<0.001
>66.6	0.11	0.08, 0.15	<0.001
Specialty			

For peer review only

General Medicine	—	—	
Toxicology	1.39	1.17, 1.65	<0.001
LOS			
<2 days	—	—	
>2 days	0.37	0.25, 0.51	<0.001

Table 2 Multiple variable logistic regression for characteristics and their association with odds of discharge against medical advice. OR = Odds Ratio, CI = Confidence Interval

The median age of death was 84 years in the PD group and 59 years in the DAMA group ($p<0.0001$ (Mann-Whitney U test)) (Table 1). Kaplan-Meier curves with stratification by age group, sex, and DAMA support this finding; showing separation of survival curves of DAMA vs. PD, with a survival advantage of planned discharge in patients under the age of 33.3, and a survival advantage of DAMA in patients over the age of 66.6 years. The stratified Kaplan Meier plots highlight that this survival advantage was restricted to those patients aged over 66.6 years on admission (Figure 1). In the younger patients, survival curves separated 2-5 years following discharge (Figure 1). The unadjusted Cox proportional hazards model suggested that DAMA offered a survival advantage (HR 2.72 95% CI(2.14-3.45)). Following adjusting for age, sex, and SIMD and including an interaction term between age and mode of discharge (to account for the fact that the risk of mortality associated with DAMA reduced with age), Cox proportional hazards models showed DAMA significantly associated with increased risk of death in the youngest patient group (aHR 2.6 95% CI(1.2-5.8)) but was protective in the older age group (aHR 0.19 95% CI(0.07-0.49)(Table 3).

Characteristic	*Simple (univariable) models*			*Multiple variable model*			*Full model with interaction*		
	HR[1]	95% CI[1]	p-value	HR[1]	95% CI[1]	p-value	HR[1]	95% CI[1]	p-value
**Discharge mode**									
Planned discharge	—	—		—	—		—	—	
DAMA	0.36	0.29, 0.47	<0.001	0.97	0.76,1.24	0.8	2.60	1.17, 5.78	0.019
**Stratified age (years)**									

For peer review only - http://bmjopen.bmj.com/site/about/guidelines.xhtml

13-33.3	—	—		—	—		—	—	
33.3-66.6	8.76	6.64, 11.5	<0.001	8.97	6.77, 11.9	<0.001	9.72	7.18, 13.1	<0.001
>66.6	55.5	42.4, 72.8	<0.001	58.9	44.7, 77.6	<0.001	64.6	48.0, 86.9	<0.001
**Sex**									
Female	—	—		—	—		—	—	
Male	0.92	0.88, 0.97	<0.001	1.04	0.98, 1.09	0.2	1.04	0.99, 1.09	0.2
**Stratified SIMD**									
<4 most deprived	—	—		—	—		—	—	
>=4 least deprived	1.23	1.16, 1.30	<0.001	0.87	0.83, 0.92	<0.001	0.87	0.83, 0.92	<0.001
**Interaction term**									
DAMA * age 33.3-66.6							0.48	0.20, 1.12	0.091
DAMA * age >66.6							0.19	0.07, 0.49	<0.001
HR = Hazard Ratio, CI = Confidence Interval									

Table 3 Cox proportional hazards models for hazard ratio of death.

The odds of death from mental illness and substance misuse, unintentional injuries and those awaiting investigation were all significantly more likely in the DAMA cohort (Table 4). These held true in after adjusting for age, sex, length of stay, specialty and 30-day readmission.

CAUSE OF DEATH	*OR*	95% CI	p
Respiratory infections (pneumonia, all viral respiratory infection including COVID, aspiration pneumonia/pneumonitis)	-0.23	-1.37, 1.24	0.71
Malignant neoplasm (all)	0.27	-0.92, 1.76	0.69
Endocrine, blood and immune disorders (excluding lymphoma/leukaemia)	-12.90	-264.74, 15.4	0.99
Mental and substance use disorders	3.97	0.76, 6.61	**0.003**
Neurological conditions (including dementia, delirium, hypoxic brain injury)	0.35	-0.99, 1.91	0.62
Cardiovascular diseases (including endocarditis, stroke, bowel ischaemia, pulmonary embolus, bleeding unspecified)	-0.42	-1.63, 1.09	0.53

For peer review only

Respiratory diseases including infective exacerbation of COPD & respiratory failure (excluding malignancy and other respiratory infection)	0.27	-1.42, 1.96	0.74
Digestive diseases including GI bleed, biliary sepsis, cholecystitis (excluding bowel malignancy & ischaemic bowel)	0.97	-0.37, 2.52	0.18
Genitourinary diseases (all renal failure & urinary tract disorder except infection)	1.01	-0.68, 2.71	0.22
Skin diseases including telangiectasia	-12.89	NA	0.99
MSK diseases (excluding infection)	-12.89	-629.78, 56.5	0.99
Unintentional injuries (burn, falls, poisoning)	3.28	1.50, 5.05	**<0.001**
Intentional injuries	-12.89	-576.03, 50.45	0.99
Old age, frailty, general decline	-0.79	-2.82, 1.01	0.39
Multi-organ failure	1.59	0.06, 3.23	0.04
Death pending investigation, unascertained	2.78	1.33, 4.39	**<0.001**

Table 4 Logistic regression of causes of death and their association with discharge against medical advice

We assessed how DAMA affected risk of readmission within 30 days of discharge. The indirect standardised incidence rate (SIR) for 30-day readmission (adjusting for age, sex, SIMD, discharge specialty, month and year of discharge and length of stay) was 1.9 (95% CI 1.5-2.2). 30-day readmission was more likely in patients under the age of 66.6 years, those who stayed in hospital <2 days at index admission, who were male, who were discharged from the toxicology unit and who were of more deprived SIMD although difference within strata was only significantly different for SIMD and discharge specialty (Figure 2).

For peer review only

DISCUSSION

This study shows in a UK-based, unselected, acute medical take, DAMA is more common in younger, male patients of more deprived SIMD. DAMA was associated with increased 30-day re-admission rates. Surprisingly, DAMA was associated with an overall reduced mortality. However, following adjustment for sex, age, SIMD and including an interaction term accounting for the reducing mortality seen in older patients who DAMA, we show that patients under the age of 33.3 years who DAMA have an adjusted HR of 2.6 for death whilst there is a survival advantage for those aged over 66.6 years who DAMA.

This study is the first to demonstrate a reduced HR for death associated with DAMA. The interaction between age and discharge mode age helps to rationalise this result as the reduced mortality was restricted to those patients over 66.6 years. Several factors may account for this finding: Older patients are likely more co-morbid and it may be more difficult for them to physically leave without assistance, thus those that were able to DAMA were possibly relatively less co-morbid than their counterparts with planned discharges. The support networks in place for the older patients who discharged against medical advice may also have been stronger than in the younger patients. The rate of DAMA in patients over 66.6 years was also much less than that seen in the younger cohort (<33.3 years 5.8% ; ≥66.6 years 0.5%) which may have selected out a specific more empowered, less frail population although we have been unable to test this hypothesis with our dataset.

The factors associated with DAMA were consistent with literature (from other healthcare systems)– young, male, and from a more deprived background (1,5,7,8,15–18). Whilst our study did not examine ethnic factors, this was noted to be a risk factor in other studies (1,5,19,20). It is unclear whether this is a unique identifier for risk of DAMA, or if there is an

For peer review only - http://bmjopen.bmj.com/site/about/guidelines.xhtml

interaction with other socioeconomic factors. In other healthcare systems, being uninsured has been associated with risk of DAMA, however we show similar rates of DAMA in a free-at-the-point-of delivery suggesting this factor is potentially less influential(9,21).

Our study demonstrates a 1.9 times increased risk of 30-day readmission in patients who DAMA. Existing literature suggests that DAMA is associated with increased hospital readmission rate with a range of 1.25 to 7 times that of the PD population depending on the specific population and presentation (16, 15, 9, 13, 19).

This is the first study of which we are aware examining DAMA in an unselected, medical cohort from the UK. We examined a large cohort over a 5-year period providing a robust analysis over an extended time frame. This is also the first study to examine DAMA in a free-at-the-point-of-delivery healthcare setting. It shows that despite free access to healthcare, 3% discharges still occur against medical advice. The temporality of the mortality rate in younger patients who DAMA, highlights that mortality rates were not noticeably different from the PD population until 2.5-5 years following discharge. This suggests that the morbidity associated with the index admission may not directly relate to the mode of death, but likely reflects a more vulnerable patient population.

There are several limitations to our study. We looked at only local, single-centre data for re-admission, meaning that if patients were readmitted to other hospitals their data was missed. We also have not been able to accurately assess co-morbidity at point of discharge, ethnicity, or how the severity of the presentation specifically affected risk of DAMA, mortality, or risk of re-admission.

For peer review only - http://bmjopen.bmj.com/site/about/guidelines.xhtml

The younger patients who discharged against medical advice died from largely preventable causes such as substance misuse, mental health disorder, and physical harm/injury. A more robust support system is clearly required for these vulnerable patients. Improved follow-up, whether taking the shape of a primary or secondary care physician, psychiatrist, social worker or substance misuse team (but ideally as a coordinated multi-disciplinary team) may provide a structure to identify and manage risk. Training and increasing awareness of the risks and context surrounding the self-discharging patient, especially amongst doctors-in-training and nursing staff may help to provide a framework for difficult conversations, appropriate escalation, referral for follow-up (both to deal with the presentation and psycho-social adversity) and, de-brief and reflection. Significant amounts of anxiety and frustration for both patients and staff often surround situations where patients elect to DAMA. Whilst often framed in a negative way, qualitative research has highlighted the strong empathy felt by the discharging team and families (22). Framing DAMA by focussing on patient empowerment and autonomy may help to guide hospital policies to focus on a patient-centred approach to DAMA; encouraging shared decision making and safe follow-up planning. Such systemic and cultural changes will likely be necessary to reduce harm for this vulnerable population of patients.

For peer review only

FIGURE LEGEND

Figure 1 - Kaplan-Meier survival curves broken down by age group and sex. Red lines show planned discharges and green lines show those against medical advice.

Figure 2 - Standardised incidence rate of 30- day readmission to hospital in DAMA cohort vs. planned discharge cohort adjusting for age, length of stay, sex, SIMD, discharge specialty, and month and year of discharge

For peer review only - http://bmjopen.bmj.com/site/about/guidelines.xhtml

For peer review only

CONTRIBUTORSHIP STATEMENT

AA: Responsible for concept and design of the work, acquisition, analysis and interpretation of data. TC: Responsible for concept and design of the work, acquisition, analysis and interpretation of data. EB: Responsible for acquisition, analysis and interpretation of data. SG: Responsible for concept and design of the work, analysis and interpretation of data. KAL: Responsible for concept and design of the work, acquisition, analysis and interpretation of data.

All authors have been involved in drafting and revision of article and are guarantors of the article and give final approval of the version to be published. They agree to be accountable for all aspects of the work in ensuring that questions related to accuracy or integrity are appropriately investigated and resolved.

For peer review only - http://bmjopen.bmj.com/site/about/guidelines.xhtml

KAL is guarantor for overall content and accepts full responsibility for the finished work and conduct of the study.

COMPETING INTERESTS

All authors declare that they have no conflicts of interest

FUNDING

This work received no specific grant from any funding agency in the public, commercial, or not-for-profit sectors.

DATA SHARING STATEMENT

No additional data available

ETHICS APPROVAL STATEMENT

This work has received Ethics approval from the local Caldicott Guardian Ethics Committee – Reference 17103

For peer review only

REFERENCES

1. Ibrahim SA, Kwoh CK, Krishnan E. Factors associated with patients who leave acute-care hospitals against medical advice. Am J Public Health. 2007 Jan 12;97(12):2204–8.

2. Spooner KK, Salemi JL, Salihu HM, Zoorob RJ. Discharge Against Medical Advice in the United States, 2002-2011. Mayo Clin Proc. 2017 Apr 1;92(4):525–35.

3. Saitz R, Ghali WA, Moskowitz MA. The impact of leaving against medical advice on hospital resource utilization. J Gen Intern Med. 2000;15(2):103–7.

4. CM W, RB M. Age contrasts in self-discharge from general hospitals. Hosp Formul. 1980 Apr 1;15(4):273, 276–7.

5. Adebowale Adefemi S. The Rate, Reasons and Predictors of Hospital Discharge against Medical Advice among Inpatients of a Tertiary Health Facility in North-Central Nigeria. Am J Heal Res [Internet]. 2015 Dec 14 [cited 2020 Jun 9];3(1):11. Available from:

For peer review only - http://bmjopen.bmj.com/site/about/guidelines.xhtml

http://www.sciencepublishinggroup.com/journal/paperinfo?journalid=656&doi=10.11648/j.ajhr.s.2015030101.12

6. Monico EP, Schwartz I. Leaving against medical advice: Facing the issue in the emergency department. J Healthc Risk Manag [Internet]. 2009 Jan 1 [cited 2020 Jun 9];29(2):6–15. Available from: http://doi.wiley.com/10.1002/jhrm.20009

7. Noohi K, Komsari S, Nakhaee N, Feyzabadi VY. Reasons for discharge against medical advice: A case study of emergency departments in Iran. Int J Heal Policy Manag. 2013;1(2):137–42.

8. Alfandre DJ. "I'm going home": Discharges against medical advice. Vol. 84, Mayo Clinic Proceedings. Elsevier Ltd; 2009. p. 255–60.

9. Hwang SW, Li J, Gupta R, Chien V, Martin RE. What happens to patients who leave hospital against medical advice? CMAJ. 2003 Feb 18;168(4):417–20.

10. Glasgow JM, Vaughn-Sarrazin M, Kaboli PJ. Leaving against medical advice (AMA): Risk of 30-day mortality and hospital readmission. J Gen Intern Med. 2010 Sep;25(9):926–9.

11. Southern WN, Nahvi S, Arnsten JH. Increased risk of mortality and readmission among patients discharged against medical advice. Am J Med. 2012 Jun;125(6):594–602.

12. Scottish Government. Scottish Index of Multiple Deprivation [Internet]. Edinburgh; 2012 [cited 2020 Nov 10]. Available from: https://www2.gov.scot/Topics/Statistics/SIMD

13. Therneau T. A Package for Survival Analysis in R. R package version 3.2-13. 2021; Available from: https://cran.r-project.org/package=survival

14. Miettinen J, Rantanen M. popEpi: Functions for Epidemiological Analysis using Population Data. R package version 0.4.8. [Internet]. Available from: https://cran.r-

For peer review only - http://bmjopen.bmj.com/site/about/guidelines.xhtml

project.org/package=popEpi

15. Baptist AP, Warrier I, Arora R, Ager J, Massanari RM. Hospitalized patients with asthma who leave against medical advice: Characteristics, reasons, and outcomes. J Allergy Clin Immunol [Internet]. 2007 Apr [cited 2021 May 22];119(4):924–9. Available from: https://pubmed.ncbi.nlm.nih.gov/17239431/

16. Jones AA, Himmelstein DU. Leaving a County Hospital Against Medical Advice [Internet]. Vol. 242, JAMA: The Journal of the American Medical Association. American Medical Association; 1979 [cited 2021 May 22]. p. 2758. Available from: https://jamanetwork.com/journals/jama/fullarticle/367919

17. Weingart SN, Davis RB, Phillips RS. Patients discharged against medical advice from a general medicine service. J Gen Intern Med [Internet]. 1998 [cited 2021 May 22];13(8):568–71. Available from: /pmc/articles/PMC1496999/?report=abstract

18. Tan SY, Tan SY, Feng JY, Feng JY, Joyce C, Fisher J, et al. Association of Hospital Discharge against Medical Advice with Readmission and In-Hospital Mortality. JAMA Netw Open [Internet]. 2020 Jun 11 [cited 2021 May 22];3(6):206009. Available from: https://jamanetwork.com/

19. Schlauch RW, Reich P, Kelly MJ. Leaving the Hospital against Medical Advice. N Engl J Med [Internet]. 1979 Jan 4 [cited 2021 May 22];300(1):22–4. Available from: https://www.nejm.org/doi/pdf/10.1056/NEJM197901043000106

20. Aliyu ZY. Discharge against medical advice: sociodemographic, clinical and financial perspectives. Int J Clin Pract [Internet]. 2002 [cited 2021 May 22];56(5):325–7. Available from: https://pubmed.ncbi.nlm.nih.gov/12137437/

21. Albayati A, Douedi S, Alshami A, Hossain MA, Sen S, Buccellato V, et al. Why do patients leave against medical advice? Reasons, consequences, prevention, and interventions. Healthc. 2021 Feb 1;9(2).

For peer review only

22. Machin LL, Goodwin D, Warriner D. An Alternative View of Self-Discharge Against Medical Advice: An Opportunity to Demonstrate Empathy, Empowerment, and Care. Qual Health Res [Internet]. 2018 Apr 1 [cited 2021 Nov 11];28(5):702–10. Available from:

For peer review only

For peer review only - http://bmjopen.bmj.com/site/about/guidelines.xhtml

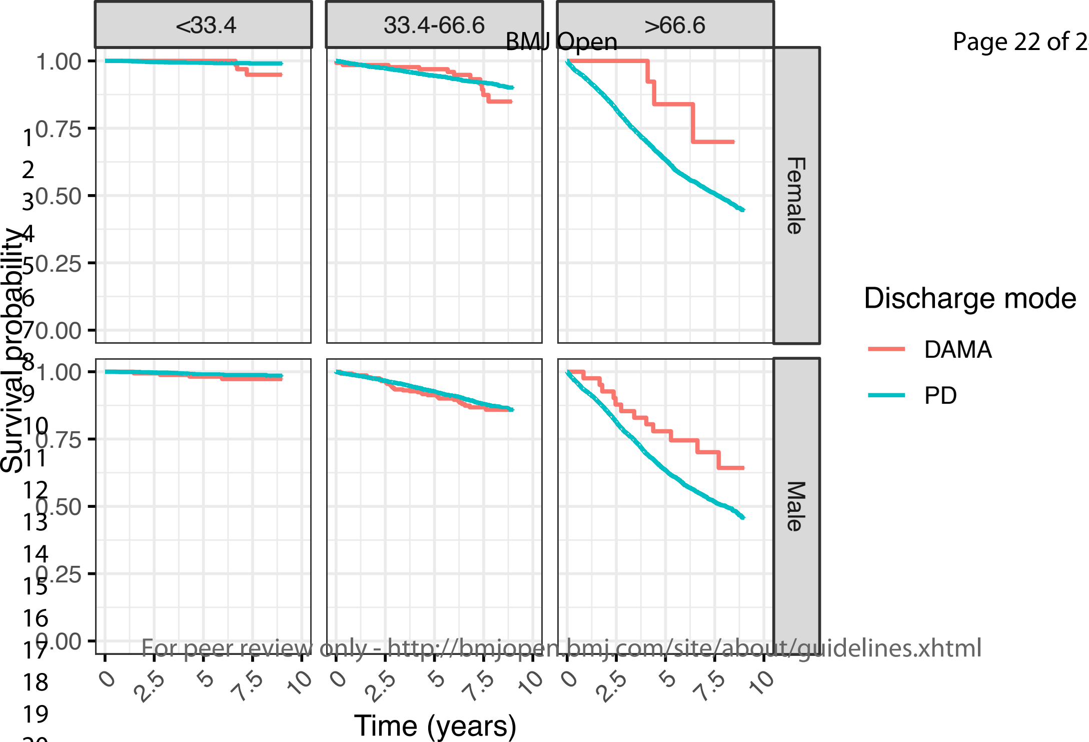

For peer review only - http://bmjopen.bmj.com/site/about/guidelines.xhtml

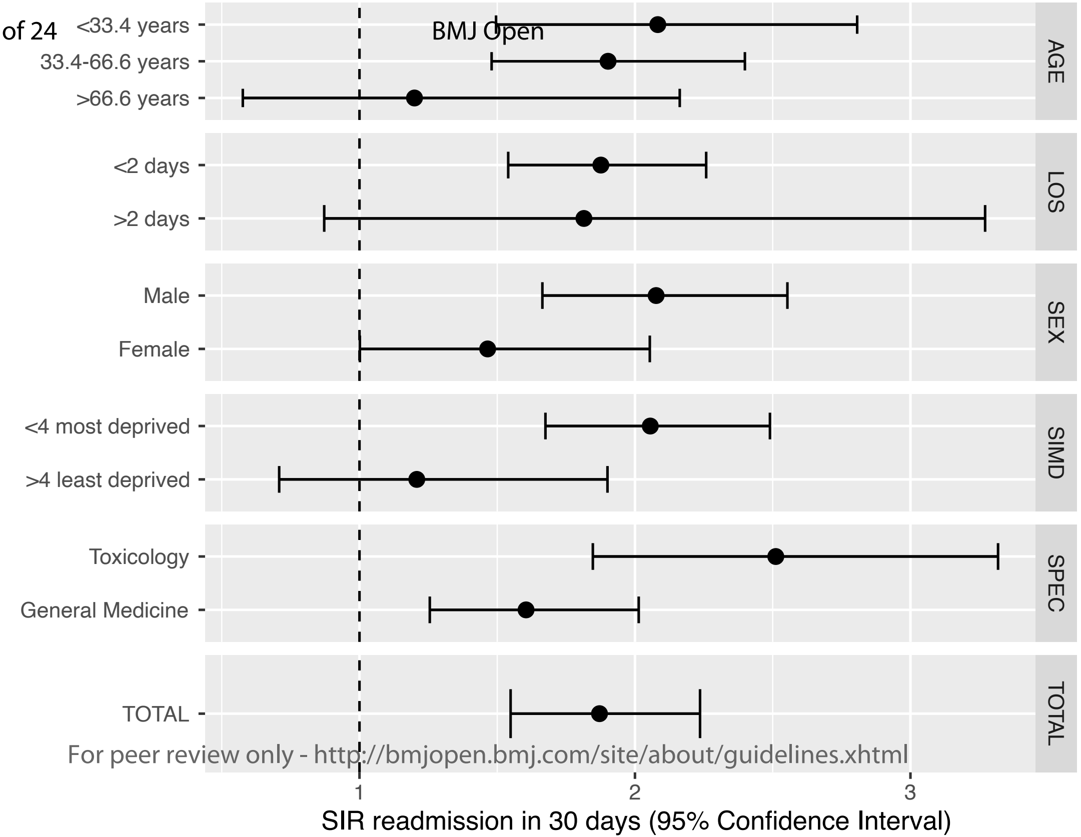

<33.4 years
33.4-66.6 years
>66.6 years
AGE
<2 days
>2 days
LOS
Male
Female
SEX
<4 most deprived
>4 least deprived
SIMD
Toxicology
General Medicine
SPEC
TOTAL
TOTAL
1
2
3
SIR readmission in 30 days (95% Confidence Interval)

STROBE Statement—Checklist of items that should be included in reports of ***cohort studies***

	Item No	Recommendation	Page No
**Title and abstract**	1	(*a*) Indicate the study's design with a commonly used term in the title or the abstract	1-2
		(*b*) Provide in the abstract an informative and balanced summary of what was done and what was found	
**Introduction**			
Background/rationale	2	Explain the scientific background and rationale for the investigation being reported	3
Objectives	3	State specific objectives, including any prespecified hypotheses	4
**Methods**			
Study design	4	Present key elements of study design early in the paper	5-7
Setting	5	Describe the setting, locations, and relevant dates, including periods of recruitment, exposure, follow-up, and data collection	5-7
Participants	6	(*a*) Give the eligibility criteria, and the sources and methods of selection of participants. Describe methods of follow-up	5-7
		(*b*) For matched studies, give matching criteria and number of exposed and unexposed	5-7
Variables	7	Clearly define all outcomes, exposures, predictors, potential confounders, and effect modifiers. Give diagnostic criteria, if applicable	5-7
Data sources/ measurement	8*	For each variable of interest, give sources of data and details of methods of assessment (measurement). Describe comparability of assessment methods if there is more than one group	5-7
Bias	9	Describe any efforts to address potential sources of bias	5-7
Study size	10	Explain how the study size was arrived at	5-7
Quantitative variables	11	Explain how quantitative variables were handled in the analyses. If applicable, describe which groupings were chosen and why	5-7
Statistical methods	12	(*a*) Describe all statistical methods, including those used to control for confounding	5-7
		(*b*) Describe any methods used to examine subgroups and interactions	
		(*c*) Explain how missing data were addressed	
		(*d*) If applicable, explain how loss to follow-up was addressed	
		(*e*) Describe any sensitivity analyses	
**Results**			
Participants	13*	(a) Report numbers of individuals at each stage of study—eg numbers potentially eligible, examined for eligibility, confirmed eligible, included in the study, completing follow-up, and analysed	7-9
		(b) Give reasons for non-participation at each stage	7-9
		(c) Consider use of a flow diagram	7-9
Descriptive data	14*	(a) Give characteristics of study participants (eg demographic, clinical, social) and information on exposures and potential confounders	7-9
		(b) Indicate number of participants with missing data for each variable of interest	7-9
		(c) Summarise follow-up time (eg, average and total amount)	7-9
Outcome data	15*	Report numbers of outcome events or summary measures over time	7-9

For peer review only - http://bmjopen.bmj.com/site/about/guidelines.xhtml

Main results	16	(*a*) Give unadjusted estimates and, if applicable, confounder-adjusted estimates and their precision (eg, 95% confidence interval). Make clear which confounders were adjusted for and why they were included	7-9
		(*b*) Report category boundaries when continuous variables were categorized	7-9
		(*c*) If relevant, consider translating estimates of relative risk into absolute risk for a meaningful time period	7-9
Other analyses	17	Report other analyses done—eg analyses of subgroups and interactions, and sensitivity analyses	7-9
**Discussion**			
Key results	18	Summarise key results with reference to study objectives	10-13
Limitations	19	Discuss limitations of the study, taking into account sources of potential bias or imprecision. Discuss both direction and magnitude of any potential bias	10-13
Interpretation	20	Give a cautious overall interpretation of results considering objectives, limitations, multiplicity of analyses, results from similar studies, and other relevant evidence	10-13
Generalisability	21	Discuss the generalisability (external validity) of the study results	10-13
**Other information**			
Funding	22	Give the source of funding and the role of the funders for the present study and, if applicable, for the original study on which the present article is based	14

*Give information separately for exposed and unexposed groups.

Note: An Explanation and Elaboration article discusses each checklist item and gives methodological background and published examples of transparent reporting. The STROBE checklist is best used in conjunction with this article (freely available on the Web sites of PLoS Medicine at http://www.plosmedicine.org/, Annals of Internal Medicine at http://www.annals.org/, and Epidemiology at http://www.epidem.com/). Information on the STROBE Initiative is available at http://www.strobe-statement.org.

For peer review only

BMJ Open

How does discharge against medical advice affect risk of mortality and unplanned readmission? A retrospective cohort study set in a large UK medical admissions unit.

Journal:	*BMJ Open*
Manuscript ID	bmjopen-2022-068801.R1
Article Type:	Original research
Date Submitted by the Author:	10-Dec-2022
Complete List of Authors:	Alagappan, Anand; Royal Infirmary of Edinburgh, Acute & General Medicine<br>Chambers, Tom; The University of Edinburgh Centre for Discovery Brain Sciences; Western General Hospital, Metabolic Unit<br>Brown, Erik; NHS Lothian, Lothian Analytical Services, Waverley Gate<br>Grecian, Sheila; Edinburgh Royal Infirmary, Diabetes and Endocrinology<br>Lockman, Khalida Ann; The University of Edinburgh, Department of Acute Medicine
**Primary Subject Heading**:	Health services research
Secondary Subject Heading:	Health informatics, Public health, Health economics
Keywords:	INTERNAL MEDICINE, PUBLIC HEALTH, Health economics < HEALTH SERVICES ADMINISTRATION & MANAGEMENT

SCHOLARONE™
Manuscripts

I, the Submitting Author has the right to grant and does grant on behalf of all authors of the Work (as defined in the below author licence), an exclusive licence and/or a non-exclusive licence for contributions from authors who are: i) UK Crown employees; ii) where BMJ has agreed a CC-BY licence shall apply, and/or iii) in accordance with the terms applicable for US Federal Government officers or employees acting as part of their official duties; on a worldwide, perpetual, irrevocable, royalty-free basis to BMJ Publishing Group Ltd ("BMJ") its licensees and where the relevant Journal is co-owned by BMJ to the co-owners of the Journal, to publish the Work in this journal and any other BMJ products and to exploit all rights, as set out in our licence.

The Submitting Author accepts and understands that any supply made under these terms is made by BMJ to the Submitting Author unless you are acting as an employee on behalf of your employer or a postgraduate student of an affiliated institution which is paying any applicable article publishing charge ("APC") for Open Access articles. Where the Submitting Author wishes to make the Work available on an Open Access basis (and intends to pay the relevant APC), the terms of reuse of such Open Access shall be governed by a Creative Commons licence – details of these licences and which Creative Commons licence will apply to this Work are set out in our licence referred to above.

Other than as permitted in any relevant BMJ Author's Self Archiving Policies, I confirm this Work has not been accepted for publication elsewhere, is not being considered for publication elsewhere and does not duplicate material already published. I confirm all authors consent to publication of this Work and authorise the granting of this licence.

For peer review only

How does discharge against medical advice affect risk of mortality and unplanned readmission? A retrospective cohort study set in a large UK medical admissions unit.

A Alagappan[1*], TJG Chambers[2,3*], E Brown[4], SM Grecian[3], KA Lockman[1]

[1]Department of Acute Medicine

The Royal Infirmary of Edinburgh and The University of Edinburgh

51 Little France Crescent, Edinburgh EH16 4SA, UK

[2]Centre for Discovery Brain Science, University of Edinburgh, Hugh Robson Building, George Square, Edinburgh, EH8 9XD

[3]Metabolic Unit, Western General Hospital, Crewe Road South, Edinburgh, EH4 2XU

[4]Lothian Analytical Services, NHS Lothian. Waverley Gate, 2-4 Waterloo Place, Edinburgh, EH1 3EG

*These authors contributed equally to this publication

Corresponding author:

Dr. Thomas Chambers- Email: tom.chambers@ed.ac.uk

For peer review only

Keywords: discharge against medical advice

For peer review only

For peer review only - http://bmjopen.bmj.com/site/about/guidelines.xhtml

ABSTRACT

Objectives: To assess the frequency of discharge against medical advice (DAMA) in a large UK teaching hospital, explore factors which increase the risk of DAMA and identify how DAMA impacts patient risk of mortality and re-admission.

Design: Retrospective cohort study.

Setting: Large acute teaching hospital in the United Kingdom.

Patients: 36,683 patients discharged from the acute medical unit of a large UK teaching hospital between January 1, 2012 and December 31, 2016.

Measurements: Patients were censored on January 1 2021. Mortality and 30-day unplanned readmission rates were assessed. Deprivation, age and sex were taken as covariates.

Results: 3% of patients discharged against medical advice. These patients were younger (median age (years) (IQR)): Planned Discharge (PD) 59 (41-77); DAMA 39 (28-51), predominantly of male sex (PD 48%; DAMA 66%), and were of greater social deprivation (in three most deprived quintiles PD 69%; DAMA 84%). DAMA was associated with increased risk of death in patients under the age of 33.3 years

For peer review only

For peer review only - http://bmjopen.bmj.com/site/about/guidelines.xhtml

(adjusted HR 2.6 (1.2-5.8)) and increased incidence of 30-day readmission (standardized incidence ratio 1.9 (1.5-2.2)).

Limitations: Re-admission to acute hospitals outside of the local health board may have been missed. We were unable to include information regarding co-morbidity or severity of presentation.

Conclusions: These data highlight the vulnerability of younger patients who DAMA, even in a free-at-the-point-of-delivery healthcare setting.

For peer review only

STRENGTHS AND LIMITATIONS

- This is the first study of which we are aware in which mortality and readmission rates are assessed in a ‘free-at-the-point of delivery’ acute medical unit
- We adjusted mortality and readmission rates for age, deprivation and sex in a dataset of 26,187 discharges from the acute medical unit
- We were not able to include information about morbidity or severity of presentation at time of index admission

For peer review only - http://bmjopen.bmj.com/site/about/guidelines.xhtml

- We show that discharge against medical advice is associated with an increase in adjusted risk of mortality and unplanned readmission, independent of the direct financial burden of healthcare
- Younger patients who discharge against medical advice highlight a vulnerable patient cohort who may benefit from intensified medical, psychological and social intervention to reduce mortality and demand for acute medical services

For peer review only

INTRODUCTION

Accounting for an estimated 1-2% of hospital discharges in the United States (1–4), discharge against medical advice (DAMA) occurs when a patient chooses to leave hospital prior to recommendation from their medical team. Of concern, the Nationwide Inpatient Sample in the US highlighted that the annual prevalence of DAMA has increased by 1.9%/annum between 2002 and 2011 (2). These figures are not unique to the United States; studies in other parts of the world show comparable figures although we could not identify examples in the literature from UK based cohorts (5–9).

Several factors associate with risk of DAMA; with a difference in perceived recovery (compared to the physician) and financial constraints thought to be most likely (2). DAMA is prevalent among vulnerable individuals, particularly those with a pre-existing diagnosis of mental health disorders and substance misuse (10,11).

Patients choosing to leave the hospital against the advice of senior clinicians are at increased risk of significant adverse outcomes. For example in previous studies in

For peer review only

the USA and Canada, patients who DAMA are up to 13.4% more likely to be re-admitted within 30 days and up to 0.6% more likely to do die within 30 days (10,11). This is thought to be related to a combination of them missing important diagnostic tests and treatment but may also reflect that underlying sociological, economic and psychological vulnerability contribute to decision making around discharge.

AIMS

We aimed to establish the contemporary incidence of DAMA in a large acute UK teaching hospital in a free-at-the-point-of-delivery health care system, identify demographic factors associated with DAMA, and to explore if DAMA was associated with risk of readmission and mortality. We considered that such data would be essential to frame revision of policies regarding the optimal management of patients choosing to discharge against medical advice.

For peer review only

METHODS

The study was approved by the local Caldicott guardian.

Study population

We conducted a retrospective analysis of all patients who were discharged from the Acute Medical Unit (AMU) at the Royal Infirmary of Edinburgh (RIE) between January 1, 2012 and December 31, 2016. The Royal Infirmary of Edinburgh serves as the largest local hospital for the region of Lothian, UK which has a population of 900,000. The RIE has a mean annual admission rate of 60,000/year and covers a geographical area of 700 square miles. The AMU is the admissions unit through which all adult medical admissions are handled. Patients are either treated and discharged directly or admitted downstream to their appropriate specialty if longer courses of treatment are required.

Data were compiled from electronic patient records; at each discharge, nursing staff document if the discharge was planned, against medical advice or was a mortality.

For peer review only

Outcomes and follow-up

The outcome measures were readmission (within 30 days) and mortality up until the point of censor on the 1st of January 2021.

Data extraction

Data was collected using TRAK (Oracle) information gathering software. The Information Services Division (ISD) of NHS Lothian provided the data linkage between the demographics and morbidity and mortality. Demographic variables included were sex, age, and Scottish Index of Multiple Deprivation (SIMD). Readmission dates were obtained from the electronic health record and mortality data obtained from linkage with National Records of Scotland (12). 3.8% of patients did not have an SIMD as no fixed address of residence was associated with the admission. These reflect patients with no fixed abode and for simplicity we included them among patients in the most deprived quintile. Age strata were defined taking into account that standardized mortality crossed unity at 36 years. We defined 3 strata giving cut offs of 33.3 and 66.6 years. Patients admitted from the accident and emergency department following accidental or intentional overdose are cohorted into

For peer review only

a toxicology bay in the acute medical unit and are given a specific toxicology code. We thus coded patients as either 'general medicine' or 'toxicology'. The STROBE checklist for

Readmissions have been identified as any patient who has an unplanned inpatient stay anywhere in NHS Lothian, in the 30 days following discharge from AMU. Inpatient stays are only counted against the most recent previous stay, so as not to count consecutive stays. Inpatient stays that may have occurred outside of NHS Lothian have not been captured.

Statistical analysis was conducted using *R* version 4.0.0. Raw data are presented with comparisons between DAMA and Planned discharge groups assessed using Pearson's Chi-square test for discrete variables and Mann Whitney U-test for continuous variables. Multiple variable logistic regression was used to assess risk factors for DAMA. Cox proportional hazards modelling for mortality was completed using the *survival* package.(13) For each covariate, we tested proportionality of hazards using Pearson product-moment correlations of Schoenfeld residuals with

For peer review only

time: we stratified age, SIMD and length of stay to minimise loss of proportionality. We chose to stratify SIMD class as <4 and >4 as the Lothian population is skewed to less deprived quintiles. Length of stay was stratified as <2 days or > 2 days given the left skew of the data (predominant short admissions). We included stratification of the Cox models by year of discharge given the trend for reducing numbers of discharges over the study period. Multiple variable logistic regression was used to assess odds of discharge against medical advice and cause of death. Indirect standardised incidence rates were calculated using *the sir* function of the *popEpi* package.(14) Significance was taken as $p<0.05$. Data are presented as medians with interquartile range.

Patient and Public Involvement

Patients or the public were not involved in the design, conduct or reporting or dissemination plans of our research

RESULTS

There were 37,409 discharges from the acute medical unit between 1st January 2012 and 31st December 2016 inclusive. We excluded 13 patients for whom we had no

For peer review only - http://bmjopen.bmj.com/site/about/guidelines.xhtml

date of birth. 10,918 discharges were excluded as they were not the first discharge for each patient within the study period; 300 were excluded as the patient had died during the admission. 26,187 first patient discharges were assessed. Of these, there were 25,430 planned discharges, and 757 DAMA (3%) (Figure 1).

For peer review only

Patients who DAMA were more likely to be male (48.1% of PD population vs. 65.8% of the DAMA population (X^2 = 92.4, p<0.0001); younger (median age 60 years (PD) vs. 39 years (DAMA); p<0.0001(Mann-Whitney U test); of more deprived SIMD (X^2 = 167.5, p <0.0001); were more likely to have an inpatient stay of under 2 days (96.3% (PD) vs 99.3% (DAMA); X^2 = 19.4, p<0.0001) and were more likely to be discharged from the toxicology unit (18.7% (PD) vs. 37.5% (DAMA); X^2 = 168.1, p <0.0001) (Table 1). The number of discharges from AMU reduced for each study year but the proportion of DAMA did not vary significantly ranging between 2.4% and 3.1%.

	PD (N=25430)	DAMA (N=757)	Total (N=26187)	p value
SEX				< 0.001
- Male (%)	12228 (48.1%)	498 (65.8%)	12726 (48.6%)	
Age at admission (years)				< 0.001
- Median (IQR)	59 (40-77)	39 (28-51)	59 (40-76)	

For peer review only - http://bmjopen.bmj.com/site/about/guidelines.xhtml

SIMD,				< 0.001
- 1 most deprived	6817 (27.2%)	310 (43.1%)	7127 (27.7%)	
- 2	5746 (23.0%)	183 (25.4%)	5929 (23.0%)	
- 3	4765 (19.0%)	112 (15.6%)	4877 (18.9%)	
- 4	4001 (16.0%)	70 (9.7%)	4071 (15.8%)	
- 5 least deprived	3697 (14.8%)	45 (6.2%)	3742 (14.5%)	
Length of stay <2 days				< 0.001
- <2 days	22058 (86.7%)	721 (95.2%)	22779 (87.0%)	
- >2 days	3372 (13.3%)	36 (4.8%)	3408 (13.0%)	
Specialty				< 0.001
- General Medicine	20626 (81.1%)	473 (62.5%)	21099 (80.6%)	
- Toxicology	4804 (18.9%)	284 (37.5%)	5088 (19.4%)	
Age of death, years				< 0.001
- n	5734	68	5802	
- Median (IQR)	84 (75-90)	59 (49-70)	84 (75-90)	

Table 1 Summary of differences in patients with planned discharge (PD) compared to those who discharge against medical advice (DAMA). Difference assessed by Chi² for categorical values, or Mann Whitney U test for continuous variables

Age under 33.3 years, male sex, a more deprived SIMD, discharge from a toxicology admission and, length of stay <2 days were all significantly associated with increased odds of DAMA as assessed by multiple variable logistic regression (Table 2).

Characteristic	OR	95% CI	p-value
SEX			
Female	—	—	
Male	1.90	1.63, 2.23	<0.001

For peer review only - http://bmjopen.bmj.com/site/about/guidelines.xhtml

SIMD 2 level			
<4 most deprived	—	—	
≥4 least deprived	0.56	0.45, 0.68	<0.001
Stratified age (years)			
13-33.3	—	—	
33.3-66.6	0.67	0.56, 0.79	<0.001
>66.6	0.11	0.08, 0.15	<0.001
Specialty			
General Medicine	—	—	
Toxicology	1.39	1.17, 1.65	<0.001
LOS			
<2 days	—	—	
>2 days	0.37	0.25, 0.51	<0.001

Table 2 Multiple variable logistic regression for characteristics and their association with odds of discharge against medical advice. OR = Odds Ratio, CI = Confidence Interval

The median age of death was 84 years in the PD group and 59 years in the DAMA group (p<0.0001 (Mann-Whitney U test)) (Table 1). Kaplan-Meier curves with stratification by age group, sex, and DAMA support this finding; showing separation of survival curves of DAMA vs. PD, with a survival advantage of planned discharge in patients under the age of 33.3, and a survival advantage of DAMA in patients over the age of 66.6 years. The stratified Kaplan Meier plots highlight that this survival

For peer review only

advantage was restricted to those patients aged over 66.6 years on admission (Figure 2). In the younger patients, survival curves separated 2-5 years following discharge (Figure 2). The unadjusted Cox proportional hazards model suggested that DAMA offered a survival advantage (HR 2.72 95% CI(2.14-3.45)). Following adjusting for age, sex, and SIMD and including an interaction term between age and mode of discharge (to account for the fact that the risk of mortality associated with DAMA reduced with age), Cox proportional hazards models showed DAMA significantly associated with increased risk of death in the youngest patient group (aHR 2.6 95% CI(1.2-5.8)) but was protective in the older age group (aHR 0.19 95% CI(0.07-0.49)(Table 3).

Characteristic	*Simple (univariable) models*			*Multiple variable model*			*Full model with interaction*		
	HR[1]	95% CI[1]	p-value	HR[1]	95% CI[1]	p-value	HR[1]	95% CI[1]	p-value
**Discharge mode**									
Planned discharge	—	—		—	—		—	—	
DAMA	0.36	0.29, 0.47	<0.001	0.97	0.76,1.24	0.8	2.60	1.17, 5.78	0.019
**Stratified age (years)**									
13-33.3	—	—		—	—		—	—	
33.3-66.6	8.76	6.64, 11.5	<0.001	8.97	6.77, 11.9	<0.001	9.72	7.18, 13.1	<0.001
>66.6	55.5	42.4, 72.8	<0.001	58.9	44.7, 77.6	<0.001	64.6	48.0, 86.9	<0.001

For peer review only - http://bmjopen.bmj.com/site/about/guidelines.xhtml

Sex									
Female	—	—		—	—		—	—	
Male	0.92	0.88, 0.97	<0.001	1.04	0.98, 1.09	0.2	1.04	0.99, 1.09	0.2
Stratified SIMD									
<4 most deprived	—	—		—	—		—	—	
>=4 least deprived	1.23	1.16, 1.30	<0.001	0.87	0.83, 0.92	<0.001	0.87	0.83, 0.92	<0.001
Interaction term									
DAMA * age 33.3-66.6							0.48	0.20, 1.12	0.091
DAMA * age >66.6							0.19	0.07, 0.49	<0.001
HR = Hazard Ratio, CI = Confidence Interval									

Table 3 Cox proportional hazards models for hazard ratio of death.

The odds of death from mental illness and substance misuse, unintentional injuries and those awaiting investigation were all significantly more likely in the DAMA cohort (Table 4). These held true in after adjusting for age, sex, length of stay, specialty and 30-day readmission.

CAUSE OF DEATH	*OR*	95% CI	p
Respiratory infections (pneumonia, all viral respiratory infection including COVID, aspiration pneumonia/pneumonitis)	-0.23	-1.37, 1.24	0.71
Malignant neoplasm (all)	0.27	-0.92, 1.76	0.69
Endocrine, blood and immune disorders (excluding lymphoma/leukaemia)	-12.90	-264.74, 15.4	0.99
Mental and substance use disorders	3.97	0.76, 6.61	**0.003**

For peer review only

Neurological conditions (including dementia, delirium, hypoxic brain injury)	0.35	-0.99, 1.91	0.62
Cardiovascular diseases (including endocarditis, stroke, bowel ischaemia, pulmonary embolus, bleeding unspecified)	-0.42	-1.63, 1.09	0.53
Respiratory diseases including infective exacerbation of COPD & respiratory failure (excluding malignancy and other respiratory infection)	0.27	-1.42, 1.96	0.74
Digestive diseases including GI bleed, biliary sepsis, cholecystitis (excluding bowel malignancy & ischaemic bowel)	0.97	-0.37, 2.52	0.18
Genitourinary diseases (all renal failure & urinary tract disorder except infection)	1.01	-0.68, 2.71	0.22
Skin diseases including telangiectasia	-12.89	NA	0.99
Musculoskeletal diseases (excluding infection)	-12.89	-629.78, 56.5	0.99
Unintentional injuries (burn, falls, poisoning)	3.28	1.50, 5.05	**<0.001**
Intentional injuries	-12.89	-576.03, 50.45	0.99
Old age, frailty, general decline	-0.79	-2.82, 1.01	0.39
Multi-organ failure	1.59	0.06, 3.23	0.04
Death pending investigation, unascertained	2.78	1.33, 4.39	**<0.001**

Table 4 Logistic regression of causes of death and their association with discharge against medical advice

We assessed how DAMA affected risk of readmission within 30 days of discharge. The indirect standardised incidence rate (SIR) for 30-day readmission (adjusting for age, sex, SIMD, discharge specialty, month and year of discharge and length of stay) was 1.9 (95% CI 1.5-2.2). 30-day readmission was more likely in patients under the age of 66.6 years, those who stayed in hospital <2 days at index admission, who were male, who were discharged from the toxicology unit and who were of more

For peer review only

deprived SIMD although difference within strata was only significantly different for SIMD and discharge specialty (Figure 3).

For peer review only

DISCUSSION

This study shows in a UK-based, unselected, acute medical take, DAMA is more common in younger, male patients of more deprived SIMD. DAMA was associated with increased 30-day re-admission rates. Surprisingly, DAMA was associated with an overall reduced mortality. However, following adjustment for sex, age, SIMD and including an interaction term accounting for the reducing mortality seen in older patients who DAMA, we show that patients under the age of 33.3 years who DAMA have an adjusted HR of 2.6 for death whilst there is a survival advantage for those aged over 66.6 years who DAMA.

This study is the first to demonstrate a reduced HR for death associated with DAMA. The interaction between age and discharge mode age helps to rationalise this result as the reduced mortality was restricted to those patients over 66.6 years. Several factors may account for this finding: Older patients are likely more co-morbid and it may be more difficult for them to physically leave without assistance, thus those that were able to DAMA were possibly relatively less co-morbid than their counterparts with planned discharges. The support networks in place for the older patients who

For peer review only

discharged against medical advice may also have been stronger than in the younger patients. The rate of DAMA in patients over 66.6 years was also much less than that seen in the younger cohort (<33.3 years 5.8% ; ≥66.6 years 0.5%) which may have selected out a specific more empowered, less frail population although we have been unable to test this hypothesis with our dataset.

The factors associated with DAMA were consistent with literature (from other healthcare systems)– young, male, and from a more deprived background (1,5,7,8,15–18). Whilst our study did not examine ethnic factors, this was noted to be a risk factor in other studies (1,5,19,20). It is unclear whether this is a unique identifier for risk of DAMA, or if there is an interaction with other socioeconomic factors. In other healthcare systems, being uninsured has been associated with risk of DAMA, however we show similar rates of DAMA in a free-at-the-point-of delivery suggesting this factor is potentially less influential(9,21).

Our study demonstrates a 1.9 times increased risk of 30-day readmission in patients who DAMA. Existing literature suggests that DAMA is associated with increased

For peer review only - http://bmjopen.bmj.com/site/about/guidelines.xhtml

hospital readmission rate with a range of 1.25 to 7 times that of the PD population depending on the specific population and presentation (16, 15, 9, 13, 19).

This is the first study of which we are aware examining DAMA in an unselected, medical cohort from the UK. We examined a large cohort over a 5-year period providing a robust analysis over an extended time frame. This is also the first study to examine DAMA in a free-at-the-point-of-delivery healthcare setting. It shows that despite free access to healthcare, 3% discharges still occur against medical advice. The temporality of the mortality rate in younger patients who DAMA, highlights that mortality rates were not noticeably different from the PD population until 2.5-5 years following discharge. This suggests that the morbidity associated with the index admission may not directly relate to the mode of death, but likely reflects a more vulnerable patient population.

There are several limitations to our study. We looked at only local, single-centre data for re-admission, meaning that if patients were readmitted to other hospitals their data was missed. We also have not been able to accurately assess co-

For peer review only - http://bmjopen.bmj.com/site/about/guidelines.xhtml

morbidity at point of discharge, ethnicity, or how the severity of the presentation specifically affected risk of DAMA, mortality, or risk of re-admission.

The younger patients who discharged against medical advice died from largely preventable causes such as substance misuse, mental health disorder, and physical harm/injury. A more robust support system is clearly required for these vulnerable patients. Improved follow-up, whether taking the shape of a primary or secondary care physician, psychiatrist, social worker or substance misuse team (but ideally as a coordinated multi-disciplinary team) may provide a structure to identify and manage risk. Training and increasing awareness of the risks and context surrounding the self-discharging patient, especially amongst doctors-in-training and nursing staff may help to provide a framework for difficult conversations, appropriate escalation, referral for follow-up (both to deal with the presentation and psycho-social adversity) and, de-brief and reflection. Significant amounts of anxiety and frustration for both patients and staff often surround situations where patients elect to DAMA. Whilst often framed in a negative way, qualitative research has highlighted the strong empathy felt by the discharging team and families (22). Framing DAMA by focussing

For peer review only

on patient empowerment and autonomy may help to guide hospital policies to focus on a patient-centred approach to DAMA; encouraging shared decision making and safe follow-up planning. Such systemic and cultural changes will likely be necessary to reduce harm for this vulnerable population of patients.

For peer review only

FIGURE LEGENDS

Figure 1-Flow chart showing the patients excluded from the initial 37,409 discharges included in initial analyses

Figure 2 - Kaplan-Meier survival curves broken down by age group and sex. Red lines show planned discharges and green lines show those against medical advice.

Figure 3 - Standardised incidence rate of 30- day readmission to hospital in DAMA cohort vs. planned discharge cohort adjusting for age, length of stay, sex, SIMD, discharge specialty, and month and year of discharge.

For peer review only - http://bmjopen.bmj.com/site/about/guidelines.xhtml

CONTRIBUTORSHIP STATEMENT

AA: Responsible for concept and design of the work, acquisition, analysis and interpretation of data. TC: Responsible for concept and design of the work, acquisition, analysis and interpretation of data. EB: Responsible for acquisition, analysis and interpretation of data. SG: Responsible for concept and design of the work, analysis and interpretation of data. KAL: Responsible for concept and design of the work, acquisition, analysis and interpretation of data.

All authors have been involved in drafting and revision of article and are guarantors of the article and give final approval of the version to be published. They agree to be accountable for all aspects of the work in ensuring that questions related to accuracy or integrity are appropriately investigated and resolved.

KAL is guarantor for overall content and accepts full responsibility for the finished work and conduct of the study.

COMPETING INTERESTS

For peer review only

All authors declare that they have no conflicts of interest

FUNDING

This work was supported by the Wellcome Trust-University of Edinburgh Institutional Support Fund (TJGC).

DATA SHARING STATEMENT

No data are available

ETHICS APPROVAL STATEMENT

This work has received Ethics approval from the local Caldicott Guardian Ethics Committee – Reference 17103

REFERENCES

1. Ibrahim SA, Kwoh CK, Krishnan E. Factors associated with patients who leave acute-care hospitals against medical advice. Am J Public Health. 2007 Jan 12;97(12):2204–8.

For peer review only

2. Spooner KK, Salemi JL, Salihu HM, Zoorob RJ. Discharge Against Medical Advice in the United States, 2002-2011. Mayo Clin Proc. 2017 Apr 1;92(4):525–35.

3. Saitz R, Ghali WA, Moskowitz MA. The impact of leaving against medical advice on hospital resource utilization. J Gen Intern Med. 2000;15(2):103–7.

4. CM W, RB M. Age contrasts in self-discharge from general hospitals. Hosp Formul. 1980 Apr 1;15(4):273, 276–7.

5. Adebowale Adefemi S. The Rate, Reasons and Predictors of Hospital Discharge against Medical Advice among Inpatients of a Tertiary Health Facility in North-Central Nigeria. Am J Heal Res [Internet]. 2015 Dec 14 [cited 2020 Jun 9];3(1):11. Available from: http://www.sciencepublishinggroup.com/journal/paperinfo?journalid=656&doi=10.11648/j.ajhr.s.2015030101.12

6. Monico EP, Schwartz I. Leaving against medical advice: Facing the issue in the emergency department. J Healthc Risk Manag [Internet]. 2009 Jan 1 [cited 2020 Jun 9];29(2):6–15. Available from: http://doi.wiley.com/10.1002/jhrm.20009

For peer review only

7. Noohi K, Komsari S, Nakhaee N, Feyzabadi VY. Reasons for discharge against medical advice: A case study of emergency departments in Iran. Int J Heal Policy Manag. 2013;1(2):137–42.

8. Alfandre DJ. “I’m going home”: Discharges against medical advice. Vol. 84, Mayo Clinic Proceedings. Elsevier Ltd; 2009. p. 255–60.

9. Hwang SW, Li J, Gupta R, Chien V, Martin RE. What happens to patients who leave hospital against medical advice? CMAJ. 2003 Feb 18;168(4):417–20.

10. Glasgow JM, Vaughn-Sarrazin M, Kaboli PJ. Leaving against medical advice (AMA): Risk of 30-day mortality and hospital readmission. J Gen Intern Med. 2010 Sep;25(9):926–9.

11. Southern WN, Nahvi S, Arnsten JH. Increased risk of mortality and readmission among patients discharged against medical advice. Am J Med. 2012 Jun;125(6):594–602.

12. Scottish Government. Scottish Index of Multiple Deprivation [Internet]. Edinburgh; 2012 [cited 2020 Nov 10]. Available from: https://www2.gov.scot/Topics/Statistics/SIMD

13. Therneau T. A Package for Survival Analysis in R. R package version 3.2-13.

For peer review only

2021; Available from: https://cran.r-project.org/package=survival

14. Miettinen J, Rantanen M. popEpi: Functions for Epidemiological Analysis using Population Data. R package version 0.4.8. [Internet]. Available from: https://cran.r-project.org/package=popEpi

15. Baptist AP, Warrier I, Arora R, Ager J, Massanari RM. Hospitalized patients with asthma who leave against medical advice: Characteristics, reasons, and outcomes. J Allergy Clin Immunol [Internet]. 2007 Apr [cited 2021 May 22];119(4):924–9. Available from: https://pubmed.ncbi.nlm.nih.gov/17239431/

16. Jones AA, Himmelstein DU. Leaving a County Hospital Against Medical Advice [Internet]. Vol. 242, JAMA: The Journal of the American Medical Association. American Medical Association; 1979 [cited 2021 May 22]. p. 2758. Available from: https://jamanetwork.com/journals/jama/fullarticle/367919

17. Weingart SN, Davis RB, Phillips RS. Patients discharged against medical advice from a general medicine service. J Gen Intern Med [Internet]. 1998 [cited 2021 May 22];13(8):568–71. Available from: /pmc/articles/PMC1496999/?report=abstract

18. Tan SY, Tan SY, Feng JY, Feng JY, Joyce C, Fisher J, et al. Association of

For peer review only

For peer review only - http://bmjopen.bmj.com/site/about/guidelines.xhtml

Hospital Discharge against Medical Advice with Readmission and In-Hospital Mortality. JAMA Netw Open [Internet]. 2020 Jun 11 [cited 2021 May 22];3(6):206009. Available from: https://jamanetwork.com/

19. Schlauch RW, Reich P, Kelly MJ. Leaving the Hospital against Medical Advice. N Engl J Med [Internet]. 1979 Jan 4 [cited 2021 May 22];300(1):22–4. Available from: https://www.nejm.org/doi/pdf/10.1056/NEJM197901043000106

20. Aliyu ZY. Discharge against medical advice: sociodemographic, clinical and financial perspectives. Int J Clin Pract [Internet]. 2002 [cited 2021 May 22];56(5):325–7. Available from: https://pubmed.ncbi.nlm.nih.gov/12137437/

21. Albayati A, Douedi S, Alshami A, Hossain MA, Sen S, Buccellato V, et al. Why do patients leave against medical advice? Reasons, consequences, prevention, and interventions. Healthc. 2021 Feb 1;9(2).

22. Machin LL, Goodwin D, Warriner D. An Alternative View of Self-Discharge Against Medical Advice: An Opportunity to Demonstrate Empathy, Empowerment, and Care. Qual Health Res [Internet]. 2018 Apr 1 [cited 2021 Nov 11];28(5):702–10.

For peer review only

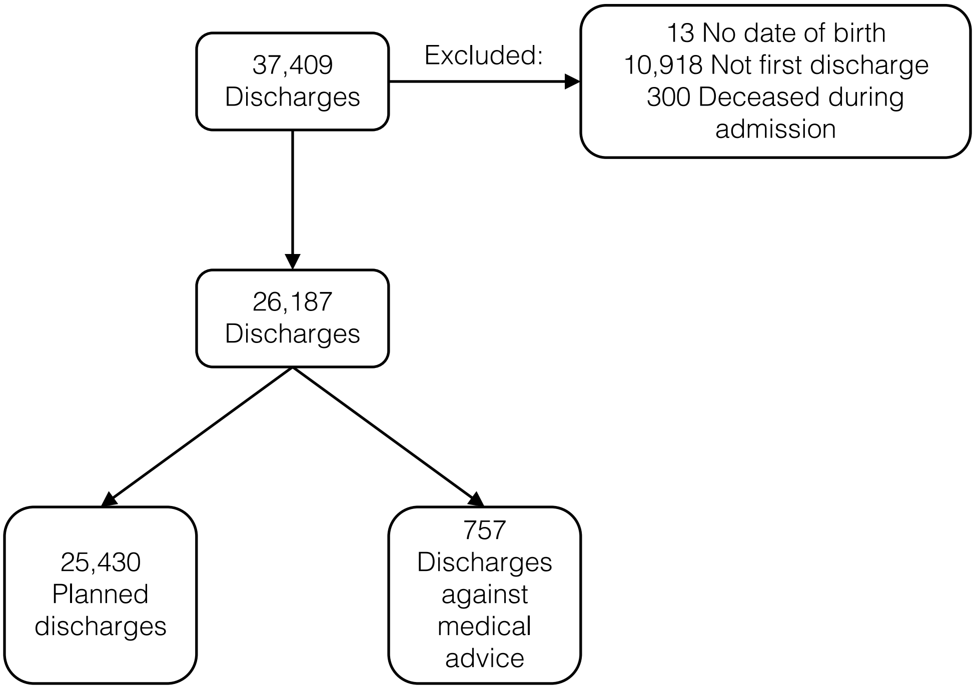
37,409
Discharges
Excluded:
13 No date of birth
10,918 Not first discharge
300 Deceased during admission
26,187
Discharges
25,430
Planned
discharges
757
Discharges
against
medical
advice

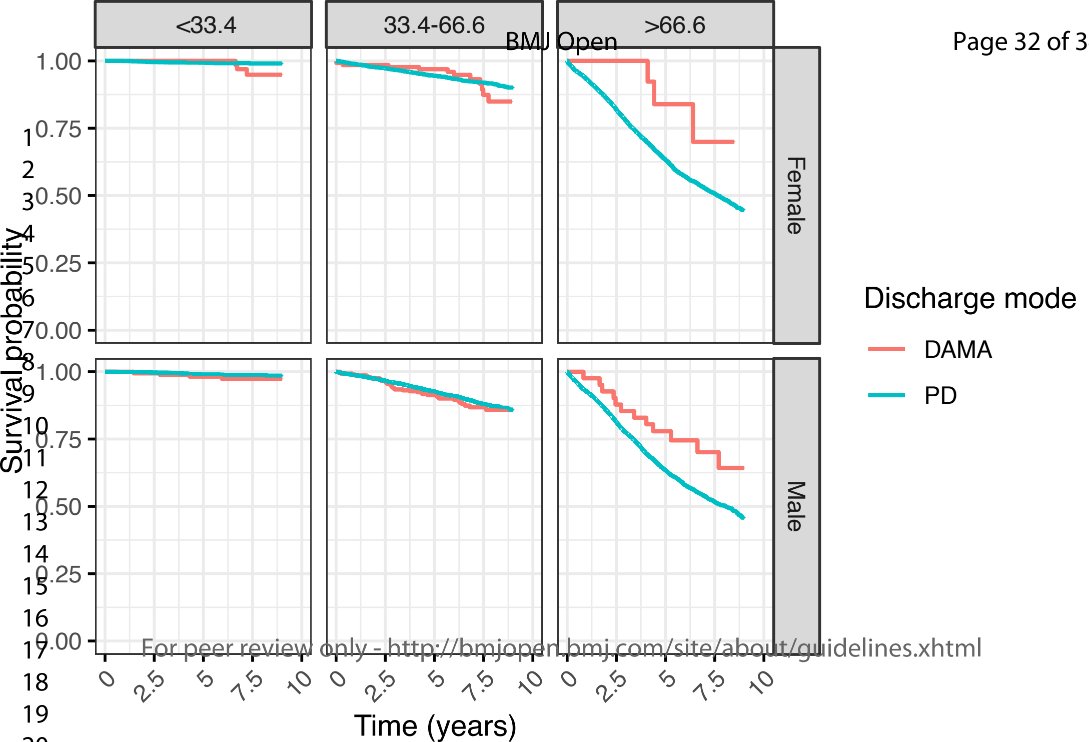

For peer review only - http://bmjopen.bmj.com/site/about/guidelines.xhtml

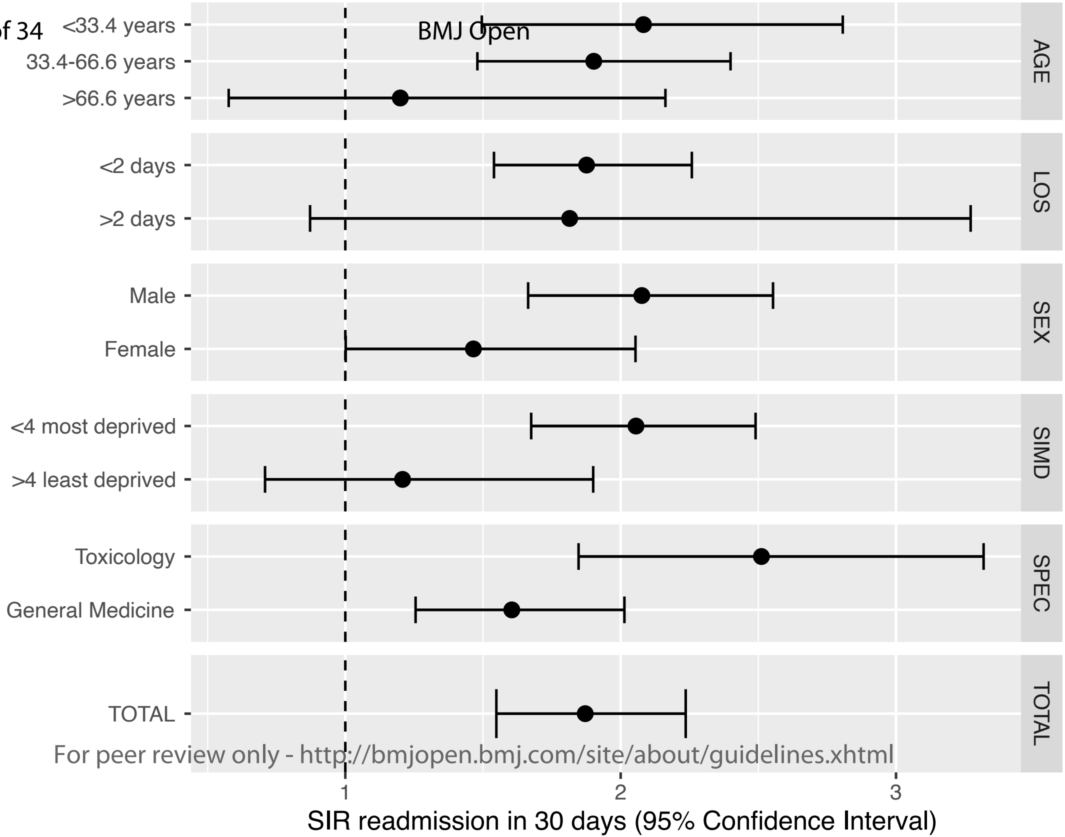

For peer review only - http://bmjopen.bmj.com/site/about/guidelines.xhtml

STROBE Statement—Checklist of items that should be included in reports of ***cohort studies***

	Item No	Recommendation	Page No
**Title and abstract**	1	(*a*) Indicate the study's design with a commonly used term in the title or the abstract	1-2
		(*b*) Provide in the abstract an informative and balanced summary of what was done and what was found	
**Introduction**			
Background/rationale	2	Explain the scientific background and rationale for the investigation being reported	3
Objectives	3	State specific objectives, including any prespecified hypotheses	4
**Methods**			
Study design	4	Present key elements of study design early in the paper	5-7
Setting	5	Describe the setting, locations, and relevant dates, including periods of recruitment, exposure, follow-up, and data collection	5-7
Participants	6	(*a*) Give the eligibility criteria, and the sources and methods of selection of participants. Describe methods of follow-up	5-7
		(*b*) For matched studies, give matching criteria and number of exposed and unexposed	5-7
Variables	7	Clearly define all outcomes, exposures, predictors, potential confounders, and effect modifiers. Give diagnostic criteria, if applicable	5-7
Data sources/ measurement	8*	For each variable of interest, give sources of data and details of methods of assessment (measurement). Describe comparability of assessment methods if there is more than one group	5-7
Bias	9	Describe any efforts to address potential sources of bias	5-7
Study size	10	Explain how the study size was arrived at	5-7
Quantitative variables	11	Explain how quantitative variables were handled in the analyses. If applicable, describe which groupings were chosen and why	5-7
Statistical methods	12	(*a*) Describe all statistical methods, including those used to control for confounding	5-7
		(*b*) Describe any methods used to examine subgroups and interactions	
		(*c*) Explain how missing data were addressed	
		(*d*) If applicable, explain how loss to follow-up was addressed	
		(*e*) Describe any sensitivity analyses	
**Results**			
Participants	13*	(a) Report numbers of individuals at each stage of study—eg numbers potentially eligible, examined for eligibility, confirmed eligible, included in the study, completing follow-up, and analysed	7-9
		(b) Give reasons for non-participation at each stage	7-9
		(c) Consider use of a flow diagram	7-9
Descriptive data	14*	(a) Give characteristics of study participants (eg demographic, clinical, social) and information on exposures and potential confounders	7-9
		(b) Indicate number of participants with missing data for each variable of interest	7-9
		(c) Summarise follow-up time (eg, average and total amount)	7-9
Outcome data	15*	Report numbers of outcome events or summary measures over time	7-9

For peer review only

For peer review only - http://bmjopen.bmj.com/site/about/guidelines.xhtml

Main results	16	(*a*) Give unadjusted estimates and, if applicable, confounder-adjusted estimates and their precision (eg, 95% confidence interval). Make clear which confounders were adjusted for and why they were included	7-9
		(*b*) Report category boundaries when continuous variables were categorized	7-9
		(*c*) If relevant, consider translating estimates of relative risk into absolute risk for a meaningful time period	7-9
Other analyses	17	Report other analyses done—eg analyses of subgroups and interactions, and sensitivity analyses	7-9
**Discussion**			
Key results	18	Summarise key results with reference to study objectives	10-13
Limitations	19	Discuss limitations of the study, taking into account sources of potential bias or imprecision. Discuss both direction and magnitude of any potential bias	10-13
Interpretation	20	Give a cautious overall interpretation of results considering objectives, limitations, multiplicity of analyses, results from similar studies, and other relevant evidence	10-13
Generalisability	21	Discuss the generalisability (external validity) of the study results	10-13
**Other information**			
Funding	22	Give the source of funding and the role of the funders for the present study and, if applicable, for the original study on which the present article is based	14

*Give information separately for exposed and unexposed groups.

Note: An Explanation and Elaboration article discusses each checklist item and gives methodological background and published examples of transparent reporting. The STROBE checklist is best used in conjunction with this article (freely available on the Web sites of PLoS Medicine at http://www.plosmedicine.org/, Annals of Internal Medicine at http://www.annals.org/, and Epidemiology at http://www.epidem.com/). Information on the STROBE Initiative is available at http://www.strobe-statement.org.

For peer review only
